# Evaluation of multiple satellite precipitation products for rainfed maize production systems over Vietnam

**DOI:** 10.1038/s41598-021-04380-8

**Published:** 2022-01-11

**Authors:** Sridhar Gummadi, Tufa Dinku, Paresh B. Shirsath, M. D. M. Kadiyala

**Affiliations:** 1 CGIAR Research Program for Climate Change, Agriculture and Food Security (CCAFS), International Rice Research Institute (IRRI), IRRI-CCAFS Office, Agricultural Genetics Institute, Km 2 Pham Van Dong Ave, Tu Liem District, Hanoi, Vietnam; 2 International Research Institute for Climate and Society, The Earth Institute at Columbia University, New York, USA; 3 CGIAR Research Program for Climate Change, Agriculture and Food Security (CCAFS, Borlaug Institute for South Asia (BISA), International Maize and Wheat Improvement Centre (CIMMYT), New Delhi, 110012 India; 4 Acharya NG Ranga Agricultural University, Guntur, India

**Keywords:** Plant sciences, Environmental sciences

## Abstract

High-resolution reliable rainfall datasets are vital for agricultural, hydrological, and weather-related applications. The accuracy of satellite estimates has a significant effect on simulation models in particular crop simulation models, which are highly sensitive to rainfall amounts, distribution, and intensity. In this study, we evaluated five widely used operational satellite rainfall estimates: CHIRP, CHIRPS, CPC, CMORPH, and GSMaP. These products are evaluated by comparing with the latest improved Vietnam-gridded rainfall data to determine their suitability for use in impact assessment models. CHIRP/S products are significantly better than CMORPH, CPC, and GsMAP with higher skill, low bias, showing a high correlation coefficient with observed data, and low mean absolute error and root mean square error. The rainfall detection ability of these products shows that CHIRP outperforms the other products with a high probability of detection (POD) scores. The performance of the different rainfall datasets in simulating maize yields across Vietnam shows that VnGP and CHIRP/S were capable of producing good estimates of average maize yields with RMSE ranging from 536 kg/ha (VnGP), 715 kg/ha (CHIRPS), 737 kg/ha (CHIRP), 759 kg/ha (GsMAP), 878 kg/ha (CMORPH) to 949 kg/ha (CPC). We illustrated that there is a potential for use of satellite rainfall estimates to overcome the issues of data scarcity in regions with sparse rain gauges.

## Introduction

Over recent decades, variability in the Asian monsoon activity poses new challenges particularly in developing countries impacting vulnerable sectors such as agriculture which significantly constitute rural livelihoods^1^. Seasonal changes in atmospheric circulation and corresponding changes in rainfall are substantially influenced by changes in the annual cycle of the Asian monsoon systems^2^. The date of onset of the Asian summer monsoon (ASM) is a key indicator for planning agricultural activities across the Asian continent which characterizes the transition from dry to wet period. Vietnam is a country with a complex topography and tropical climate^3,4^, the monsoon climatology of Vietnam is greatly influenced by the South Asian, East Asian, and Australian monsoon systems^5^. The south Asian summer monsoon (SASM) and the East Asian winter monsoon (EAWM) are two distinct Asian monsoons that regulate rainfall activity over Vietnam. Major rainfall appears in the northern part of Southeast Asia during the summer (JJAS) monsoon season^6–8^. The wet season is characterized from early May to mid-October, whereas November to late April represents the dry season. A clear transition from the dry to the wet season is illustrated by rainfall received in late April. The seasonal transition of the atmospheric circulation over mid and low latitudes due to the rapidly warming landmass of Asia is the physical mechanism responsible for the SASM onset^9,10^. Climate variability over Vietnam may negatively impact many socio-economic sectors which are highly dependent on monsoon activity such as forestry, fisheries, and agriculture. Therefore, it is of utmost importance to understand the dynamics of the monsoons and their impacts on the country’s socio-economic sectors. To understand the possible association of agricultural performance in a variable climate, analysis of rainfall variability and trends requires long-term spatial rainfall time-series. To comprehend the potential impacts of climate variability and change requires long-term high-resolution climate time series. In general, rainfall measurements from ground meteorological stations are the principal sources of such data. Normally, rain gauges provide accurate point measurements of precipitation, however, historical records from station observations are insufficient over many parts of the world due to sparse station networks. On numerous occasions across the globe, satellite rainfall products have been used successfully to construct precipitation information derived from satellite observations of infrared (IR) and microwave (MW) radiance^11,12^. Space-borne measurements of precipitation have produced operational precipitation products based on satellite observations of infrared^13–15^ and passive microwave^16–18^ with continuous evolution and refinements of retrieval algorithms. Combining information from multiple satellite sensors with observed rain gauge and numerical climate model outputs has improved global precipitation datasets^15,19,20^. Thus, merged satellite-based rainfall products with station observations have been increasingly used for modeling studies. In recent years long-term satellite rainfall provides a detailed assessment of rainfall climate for a given region. These include the Climate Hazards Group (CHG) Infrared Precipitation (CHIRP) and CHIRP combined with station data CHIRPS from the University of California at Santa Barbara and U.S. Geological Survey^21,22^; the Global Precipitation Climatology Project^23^; the Climate Prediction Centre (CPC) Merged Analysis of Precipitation^15^; Tropical Applications of Meteorology using SATellite and ground-based observations rainfall estimate (TAMSAT)^24–27^ and the African Rainfall Climatology version 2^28^.

Precipitation in many environments is a highly variable and contributing factor to agricultural systems. The Spatio-temporal variability of precipitation has received considerable attention from researchers in recent decades due to greater reliance on climate-sensitive sectors, particularly agriculture. Rainfall variability is a global phenomenon, its impacts on vulnerable sectors such as agriculture are strongest, particularly in developing countries. The sustainable socio-economic development of Vietnam still largely depends on agricultural activities. Agriculture contributes 18.39% of GDP^29^, ensures national food security, and exports several major agricultural products (rice, coffee, rubber, etc.). Increasing incidences of extreme weather events such as floods^30,31^ and cold spells^32,33^ in the north and north-central coast, saltwater intrusion in the Mekong River Delta^34,35^, and droughts in the Central Highlands^36–38^, have shown that climate variability and change is becoming more apparent in Vietnam. To maintain agricultural production under increasing climate risk, it is, therefore, important to understand how precipitation and surface temperatures are varying over Vietnam in space and time. Such vulnerability assessment can serve as a framework for the identification of susceptible sectors and further develop strategic adaptation plans. Monitoring climate-related hazards are considered as one of the highest priorities of the National Adaptation Programmes of Action of least developed countries, particularly in Vietnam. Accurate and reliable precipitation records are crucial, not only to investigate the spatial pattern and temporal change of precipitation but also to improve the accuracy of agricultural simulation^39,40^. Few studies to date have evaluated the satellite rainfall products over Vietnam. The most relevant^41–43^, evaluated the performance of satellite rainfall products such as GsMAP, CMORPH, TRMM 3B42 over different regions of Vietnam.

Crop Simulation Models allow to represent crop growth, development, and yields to evaluate^44^ new technologies, quantify climate-related risk in current and future climates. Crop risk assessment in a changing environment can be estimated through long-term crop simulations^43^, which requires historical daily weather data that many times are not available. Alternatively, gridded climate datasets are available, such as derived climate data from global/regional climate models, interpolated observed stations data, and satellite estimated precipitation products. The success of crop simulation studies strongly depends on the input datasets such as daily precipitation, surface temperatures, soil, and cultivar genetic coefficients. The spatial and temporal variability of weather conditions is an important source of uncertainty when applying crop simulation models over large areas. The current study aims to evaluate five satellite estimated rainfall over Vietnam and applying a crop simulation model on a regional scale to evaluate and quantify the uncertainty that arises in estimating maize yield using Decision Support System for Agrotechnology Transfer (DSSAT) model, when observed weather data (precipitation), are replaced with satellite products.

## Results

### Validation of annual and seasonal rainfall totals

**Figure 1:**
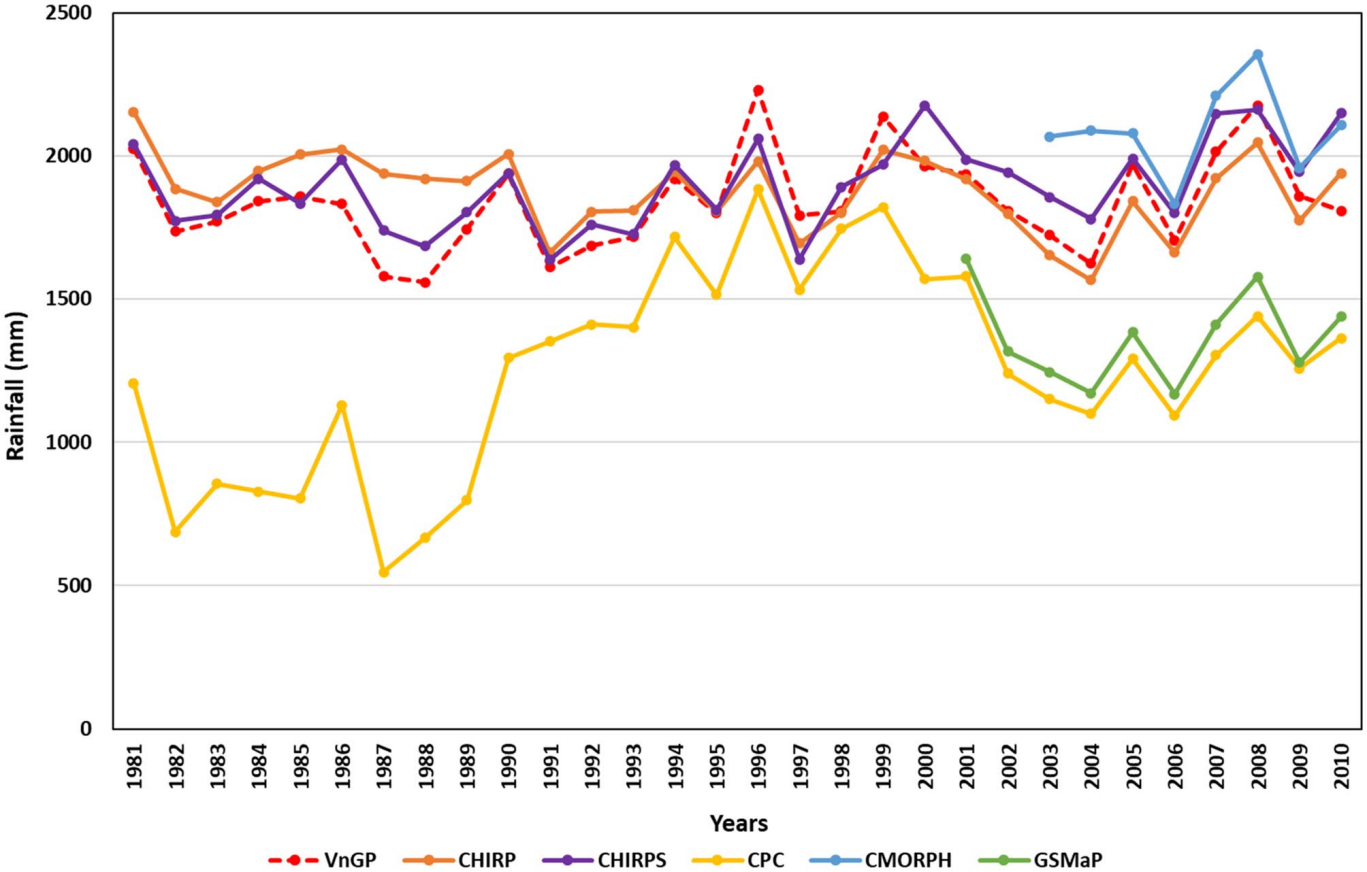
Comparison of area-weighted annual rainfall over Vietnam during 1981–2010.

**Figure 2:**
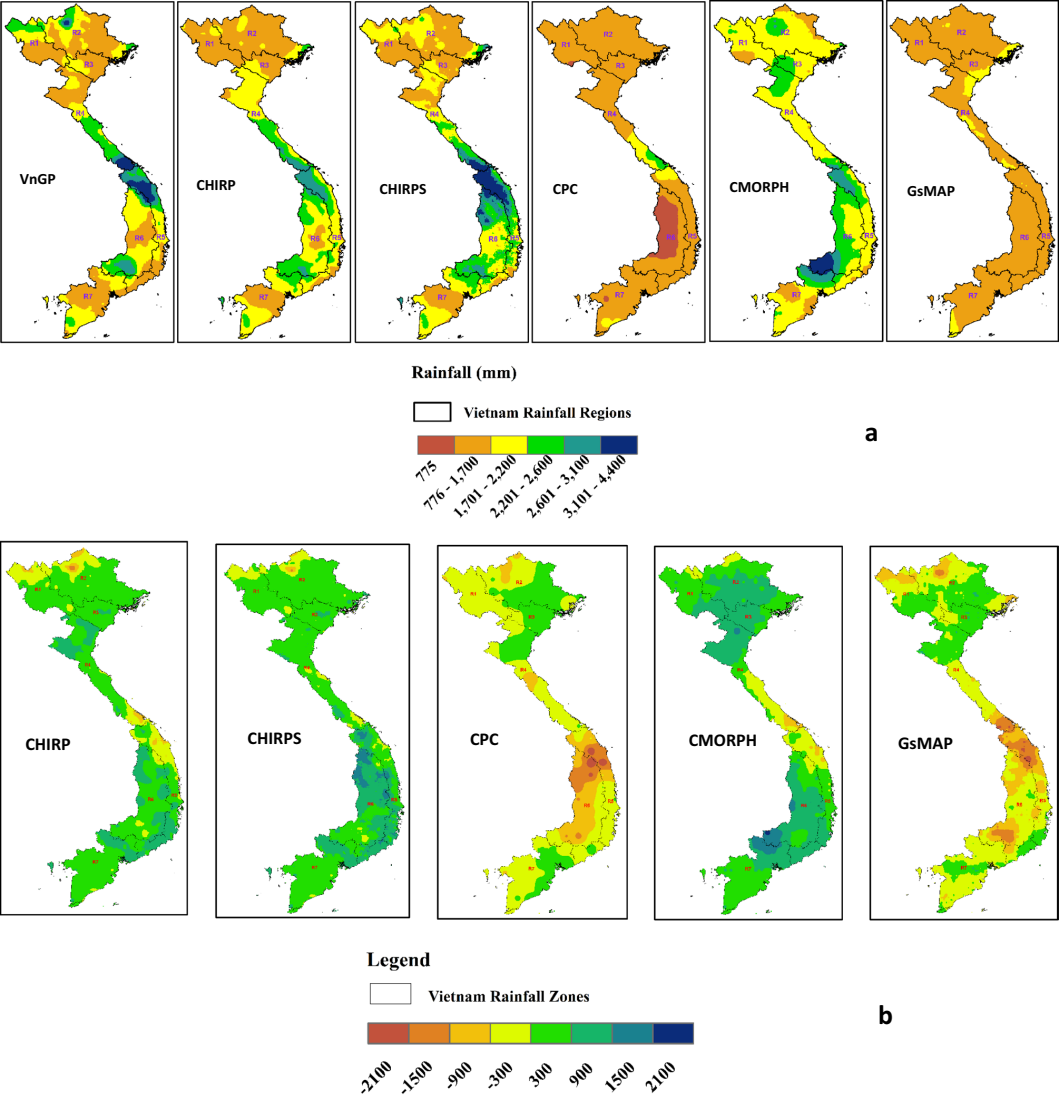
(**a**) Spatial distribution of mean Annual rainfall over Vietnam, comparing observed rainfall totals with satellite rainfall products (**b**) Spatial distribution of mean Annual difference between observed rainfall (VnGP) and satellite estimated rainfall products over Vietnam. These maps were generated using ArcGIS 10.8.1 (https://www.arcgis.com/index.html).

Validation statistics were calculated for each grid point and aggregated to rainfall regions (R1-R7) as well as a national scale. Satellite rainfall products and observed gridded rainfall (VnGP) data are available for different periods. Hence, the overlapping period 1981–2010 was selected for the current work. Since VnGP data are available from 1981 to 2010, a comparison of CHIRP/S and CPC is performed for the period 1981 to 2010, while CMORPH and GSMaP, a comparison is performed during 2003 to 2010 and 2001 to 2010 respectively. Accuracy assessment of rainfall totals of satellite estimated rainfall products is performed at the national and regional scale, area-weighted annual rainfall for Vietnam (Fig. 1) for the observed and satellite products exhibited that CHIRP/S and CMORPH are overestimating throughout the study period while CPC and GsMAP are underestimating. However, estimated rainfall products were able to capture the interannual variability. The observed annual rainfall totals over Vietnam varied from 1582 mm/year at R3 to 2208 at R5 and the coefficient of variation (CV %) varied from 9.7 percent at R7 to 21.2 percent at R5 as represented in Table 1. The estimated rainfall datasets differed in their accuracy to estimate the amount of rainfall received, both CHIRP and CHIRPS estimated rainfall amounts are within ± 15% of the observed rainfall amounts at a regional scale. However, at R3, R6, and R7 both CHIRP and CHIRPS are overestimating rainfall totals (CHIRP: R3 (10.15%), R6 (8.2%), R7 (6.5%); CHIRPS: R3 (6.7%), R6 (12.9%), R7 (4.3%)). CPC and GsMAP estimations are way below the observed rainfall totals and vary from − 53 to – 0.8%, CMORPH tends to overestimate rainfall totals particularly at R3 (31%), R6 (25%), and R7 (16%), in the rest of the rainfall regions CMORPH performance is to some extent better with a ± 10% difference. CHIRP/S datasets displayed very low interannual variability across Vietnam. While, CMORPH, CPC, and GsMAP rainfall products displayed the highest interannual variability in annual rainfall totals across Vietnam. A close estimate of annual rainfall totals spatially is observed with CHIRP/S and CMORPH (Fig. 2).

**Table 1:** Observed and satellite estimated annual rainfall totals (mm) along with the coefficient of variation at different rainfall regions over Vietnam during 1981–2010.

Data source	R1	R2	R3	R4	R5	R6	R7
**VnGP**							
Mean (mm)	1772.7	1858.6	1582.8	2030.0	2208.4	1963.7	1728.9
CV (%)	11.2	10.6	16.4	12.4	21.2	10.6	9.7
**CHIRP**							
Mean (mm)	1700.9	1724.1	1743.5	1873.1	2117.6	2126.1	1842.7
CV (%)	8.9	12.3	11.6	13.1	9.1	9.3	9.4
**CHIRPS**							
Mean (mm)	1646.8	1707.6	1690.1	1918.2	2217.0	2217.6	1803.1
CV (%)	13.3	12.7	12.6	17.5	13.0	11.2	11.7
**CPC**							
Mean (mm)	1305.6	1275.4	1233.4	1426.9	1246.5	925.4	1357.8
CV (%)	15.6	11.6	30.6	31.8	42.2	58.3	33.7
**CMORPH**							
Mean (mm)	1934.3	1947.9	2079.1	2108.9	2088.8	2445.8	2006.3
CV (%)	17.3	21.7	26.6	26.3	27.2	20.4	16.6
**GSMaP**							
Mean (mm)	1282.2	1392.4	1581.4	1577.5	1331.7	1010.0	1368.0
CV (%)	16.1	18.4	22.3	15.1	26.9	18.0	9.0

The accuracy of these satellite products to reproduce observed rainfall was investigated at seasonal time scales. Seasonal rainfall has a significant influence on agricultural activities, particularly in predominant rainfed agricultural systems. Vietnam receives 54 percent of the annual rainfall during JJAS months in most of the rainfall regions, except over the central Vietnam (R4 and R5), where the major rainfall occurs during September–November. The average observed JJAS rainfall over Vietnam is 986 mm during the 1981–2010 period, with both CHIRP and CHIRPS datasets overestimating JJAS rainfall by 14 percent (1126 and 1121 mm). CPC estimated rainfall is underestimating seasonal rainfall totals by -32% (670 mm), while, CMORPH is overestimating JJAS rainfall by 31 percent (1292 mm) during the period 2003–2010. GSMaP estimated rainfall amounts during the JJAS period during 2001–2010 is 870 mm which is 12% percent less than observed JJAS totals. The highest annual rainfall amounts were observed over central Vietnam (R4 and R5) which is received during September–November months. CHIRP/S and CMORPH estimated the highest rainfall at R4 and R5, however, CHIRP/S and CMORPH underestimated annual rainfall amounts over the R4 and R5 regions compared with VnGP rainfall amounts. CHIRPS precisely captured the spatial patterns of annual rainfall distribution followed by CHRIP and CMORPH as displayed in Fig. 2.

### Validation of satellite data using categorical statistics

**Figure 3:**
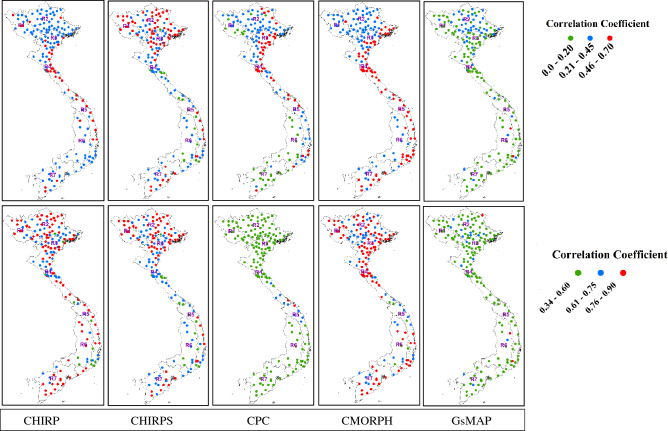
Spatial distribution of correlation coefficient (r) between rain gauge station and satellite rainfall at daily (upper) and dekadal (lower) scale. These maps were generated using ArcGIS 10.8.1 (https://www.arcgis.com/index.html).

Rainfall estimates for the five satellite products (i.e., CHIRP, CHIRPS, CPC, CMORPH, and GSMaP) were compared with the point-pixel approach and averaged over a range of rainfall regions in Vietnam at dekadal (10-day) time scales (Table 2). The computed coefficient of correlation between satellite estimates and observed rainfall across Vietnam showed a weak linear relationship at daily time scales (0.20 ≤ r ≤ 0.64) as compared to the positive and strong linear relationship at the dekadal time scales (0.53 ≤ r ≤ 0.90) for all the satellite products. Although the satellite products were observed to produce a weak correlation with observed data at the daily time scale across the rainfall regions (Fig. 3), further analysis showed an improved relationship at the dekadal and monthly time scale. As depicted in Fig. 3, the strongest correlation between daily observation and satellite rainfall data at national scale is observed for CPC (Avg: 0.52; Min: 0.20 and Max: 0.77), followed by CMORPH (Avg: 0.44; Min: 0.28 and Max: 0.62), CHIRP (Avg: 0.41; Min: 0.28 and Max: 0.55), CHIRPS (Avg: 0.40; Min: 0.23 and Max: 0.53), and GSMaP (Avg: 0.23; Min: 0.20 and Max: 0.57) in that order. Similarly, the strongest correlation coefficient between dekadal observed rainfall and satellite rainfall at national scale is detected for CMORPH (Avg: 0.77; Min: 0.47 and Max: 0.88), followed by CHIRP (Avg: 0.74; Min: 0.34 and Max: 0.86), CHIRPS (Avg: 0.73; Min: 0.31 and Max: 0.85), CPC (Avg: 0.53; Min: 0.20 and Max: 0.80) and GSMaP (Avg: 0.36; Min: 0.20 and Max: 0.76) in that order.

**Table 2:** Validation statistics of satellite-based rainfall data dekadal time scales for different rainfall regions of Vietnam.

Source	Quantitative statistics	R1	R2	R3	R4	R5	R6	R7
CHIRP	MAE	28.35	29.57	28.57	28.42	26.58	27.45	28.54
	RMSE	46.89	52.71	50.52	49.70	44.14	47.62	51.66
	NSCE	0.71	0.76	0.75	0.78	0.79	0.74	0.71
	Bias	0.90	0.88	0.97	0.93	0.86	0.92	0.96
CHIRPS	MAE	29.58	31.33	29.92	29.39	25.94	28.23	29.45
	RMSE	48.92	54.88	51.63	50.17	43.18	47.89	51.46
	NSCE	0.76	0.70	0.71	0.76	0.80	0.73	0.70
	Bias	0.95	0.92	0.98	0.94	0.91	0.94	0.98
CPC	MAE	37.86	40.24	39.96	38.28	36.95	35.80	36.93
	RMSE	54.10	64.45	65.27	59.40	52.79	57.27	60.47
	NSCE	0.48	0.33	0.43	0.45	0.56	0.57	0.58
	Bias	0.54	0.52	0.58	0.60	0.54	0.50	0.60
CMORPH	MAE	38.72	38.48	39.86	37.64	35.34	37.25	36.44
	RMSE	66.27	71.12	73.27	68.99	61.74	67.53	70.68
	NSCE	0.66	0.66	0.70	0.79	0.81	0.80	0.71
	Bias	0.66	0.71	0.60	0.55	0.77	0.72	0.85
GSMaP	MAE	49.72	50.68	53.14	50.55	48.74	50.85	50.87
	RMSE	81.93	88.71	92.13	86.65	80.95	87.31	90.43
	NSCE	0.54	0.59	0.62	0.62	0.72	0.63	0.61
	Bias	0.50	0.48	0.51	0.60	0.55	0.52	0.63

The performance of the satellite products in reproducing rainfall in different rainfall regions at the daily time scale is varying across satellite products. The MAE for CHIRP varied from 26.6 mm at R5 to 29.6 mm at R2, similarly, for CHIRPS MAE values ranged from 25.9 mm at R5 to 31.3 mm at R2. Highest MAE values are noticed for CPC ranged from 35.8 mm at R6 to 40.2 mm at R2. CMORPH too exhibited higher MAE values ranging from 35.3 mm at R5 to 39.9 mm at R3, GSMaP too exhibited the highest MAE values varying from 48.7 mm at R5 to 53.14 mm at R3. The spatial patterns of RMSE for specific rainfall products are varying throughout the study period over Vietnam. CHIRP/S is superior to the other rainfall products, with the lowest RMSE for CHIRP (lowest at R5 with 44.1 mm/dekadal and highest at R2 with 52.7 mm/dekadal) followed by CHIRPS (lowest at R5 with 43.2 mm/dekadal and highest at R2 with 54.8 mm/dekadal), CPC (52.8 at R5 and 65.3 at R3), CMORPH (61.7 at R5 and 73.3 at R3), and GSMaP (81.0 at R5 and 92.1 at R3) in that order. The NSCE values for different rainfall products at national scale were as follows: 0.75 for CHIRP (lowest at R1 and R7 (0.71) and highest at R4 and R5 (0.79)), 0.74 for CHIRPS (lowest at R2 and R7 (0.70) and highest at R5 (0.80)), 0.73 for CMORPH (lowest at R1 and R2 (0.66) and highest at R5 and R6 (0.81)), 0.62 for GsMAP (lowest at R1 (0.54) and highest at R5 (0.72)) and 0.49 for CPC (lowest at R2 (0.33) and highest at R7 (0.58)). While the NSCE values for the CHIRP, CHIRPS, and CMORPH indicating good skill in reproducing rainfall volume, the low NSCE values for GsMAP and CPC indicating that the predicted rainfall was not as good as other rainfall products used in the study.

**Figure 4:**
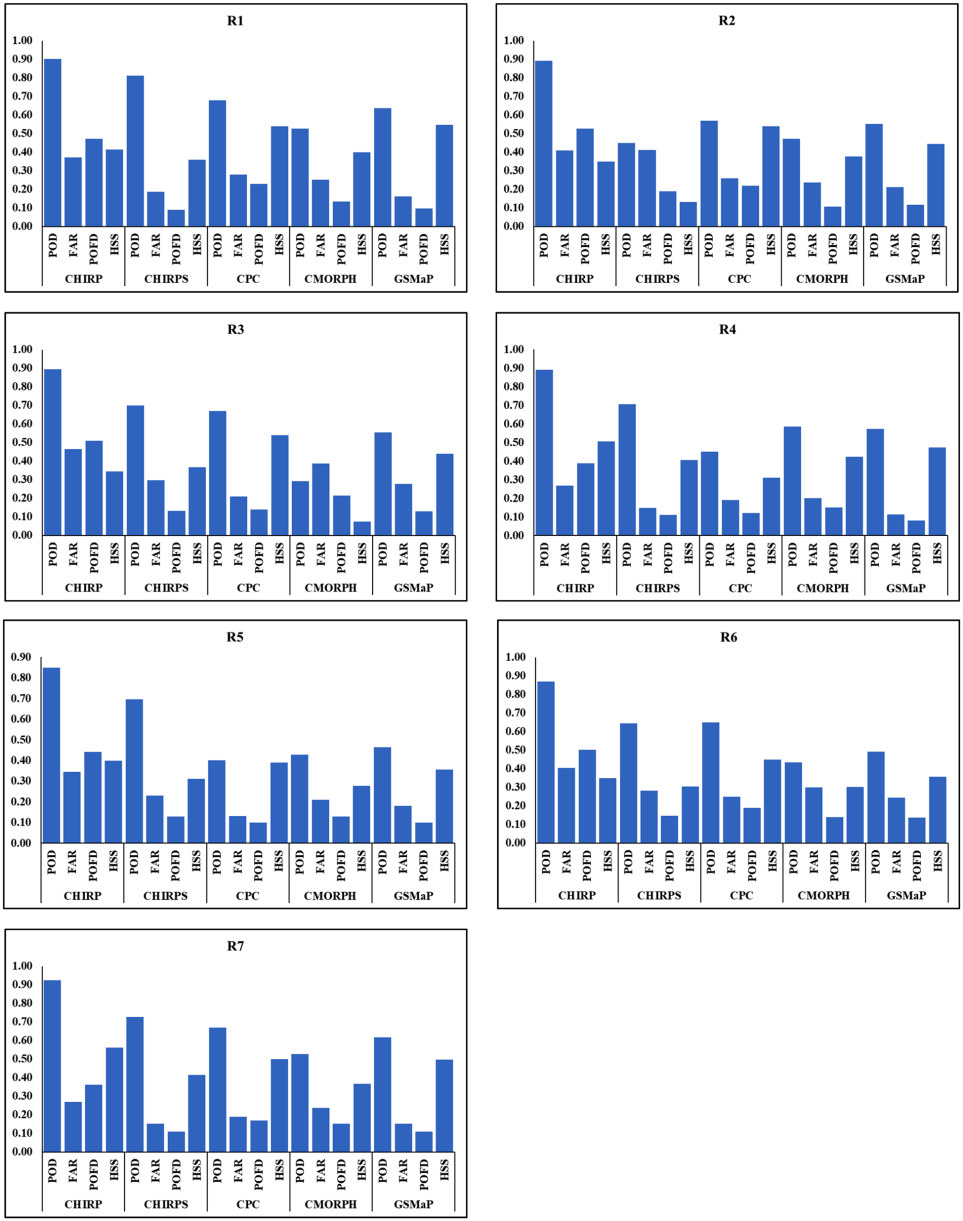
Performance of different satellite rainfall products over Vietnam using different validation statistics. Where POD is Probability of Detection, FAR is False Alarm Ratio, POFD is Probability of False Detection and HSS is Heidke Skill Score.

The categorical statistics for the five satellite products evaluated over entire Vietnam are presented in Fig. 4. It is observed that, in general, POD for CHIRP is higher across all the rainfall regions followed by CHIRPS, CPC, CMORPH, and GSMAP. Both CMORPH and GsMAP performance is relatively poor over Vietnam, POD of all the satellite products is relatively poor over the R2 and R5 rainfall regions except for CHIRPS. The lowest value is observed for CMORPH. The POD values for CHIRP are consistent across different rainfall regions of Vietnam varying from 85% at R5 to 92% at R7, CHIRPS displayed the lowest value at R2 (45%) while CPC values across Vietnam are varying from 40% at R5 to 68% at R1. GSMaP on the other hand displayed the lowest values at R5 (46%) and R4 (49%). CHIRP outperforms the other products in terms of rainfall detection capability with a mean POD of 89% across Vietnam. The FAR values are very low for all the products varying from 0.19 for GsMAP to 0.36 for CHIRP. The lowest FAR value is observed for GSMaP (average FAR value across 7 regions is 0.19). These statistics show that satellite products were able to detect rainfall when it occurs and that they detect rain that does not reach the ground. FAR value for CHIRP is varying from 0.27 at R6 and R7 to 0.46 at R3, while for CHIRPS, FAR values range from 0.15 at R6 and R7 to 0.41 at R2. FAR values for CPC are relatively low and fluctuate from 0.19 at R6 and R7 to 0.28 at R1. CMORPH on the other hand displayed FAR values differing from 0.20 at R6 to 0.39 at R3. The lower FAR values indicate that satellite rainfall products reasonably good at detecting the low possibility of incorrectly no-rain events as rain events. POFD displays the ratio of false alarms to the total no-rain events, lowest POFD will be better if it gets closer to zero. GSMaP obtained lowest POFD value (11%) followed by CHIRPS (13%), CHMORPH (15%), CPC (17%), and CHIRP (46%). The HSS values for CHIRP varied from 0.34 at R3 to 0.56 at R7, similarly the HSS value for CHIRPS spatially varied from 0.13 at R2 to 0.41 at R6 and R7. GsMAP displayed the highest HSS score across different rainfall regions of Vietnam and differed between 0.36 at R4 and R5 and 0.55 at R7, CMORPH on the other hand showed that HSS values ranged between 0.07 at R3 to 0.42 at R6. The HSS values again show that the skill of the satellite products in detecting rainfall occurrences is much better than random chance. The results of the categorial scores show good rainfall detection ability, both CHIRP (POD: 0.89) and CHIRPS (POD:0.68) scores indicates best performance over Vietnam. The low FAR values of CPC (0.22) and CHIRPS (0.24) out performs all the satellite products in the region, FAR shows the ratio of falsely detected events to the total detected events. Over all the categorial scores indicate CHIRPS is the best performing followed by CHIRP, CMORPH, GsMAP and CPC.

### Crop models validation

**Figure 5:**
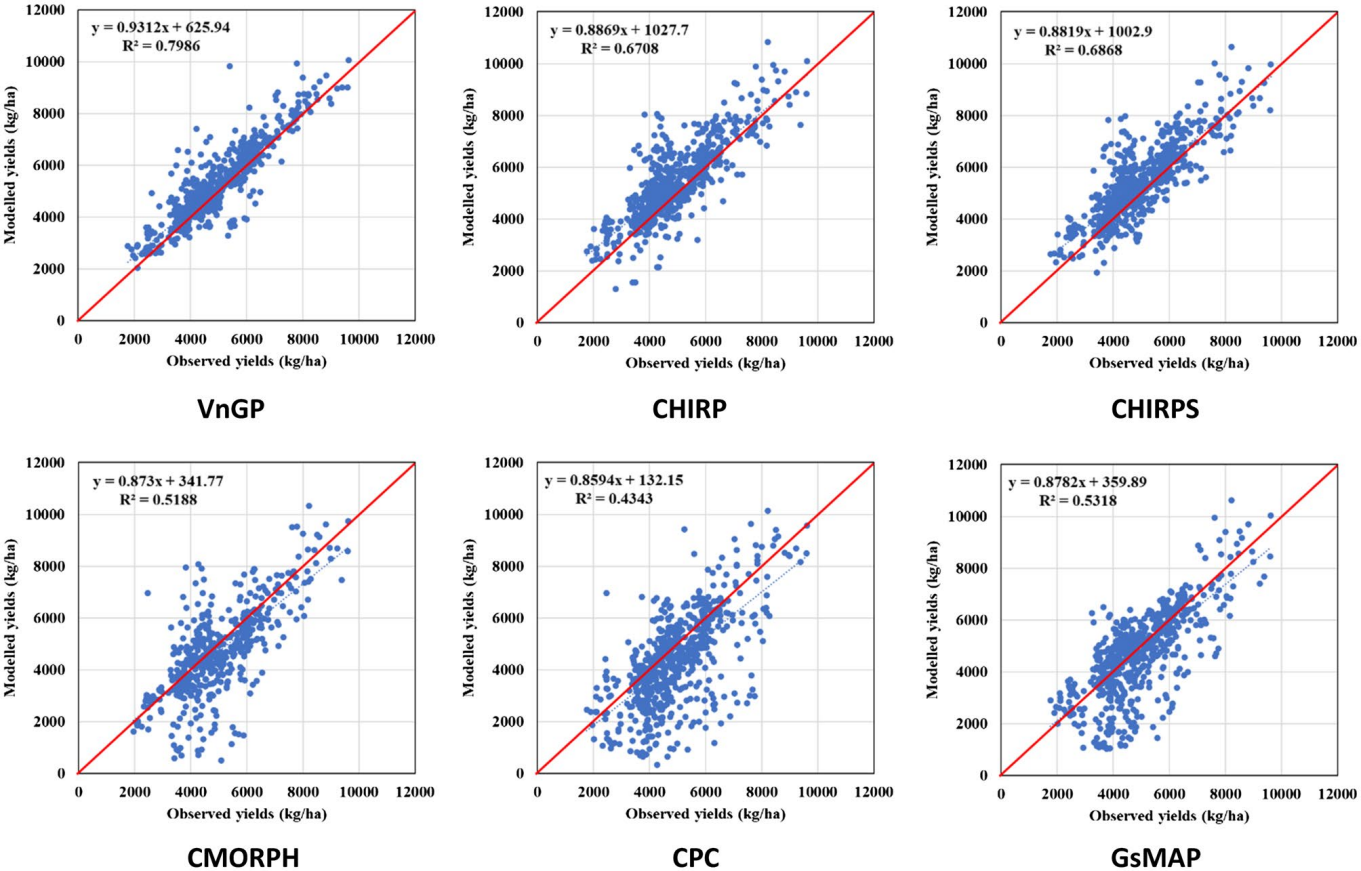
Relationship between observed (GSO maize yield data) and simulated maize yields for Vietnam. Solid red line shows 1:1 relationship, dotted line represents the linear relationship between observed and simulated crop yields.

**Figure 6:**
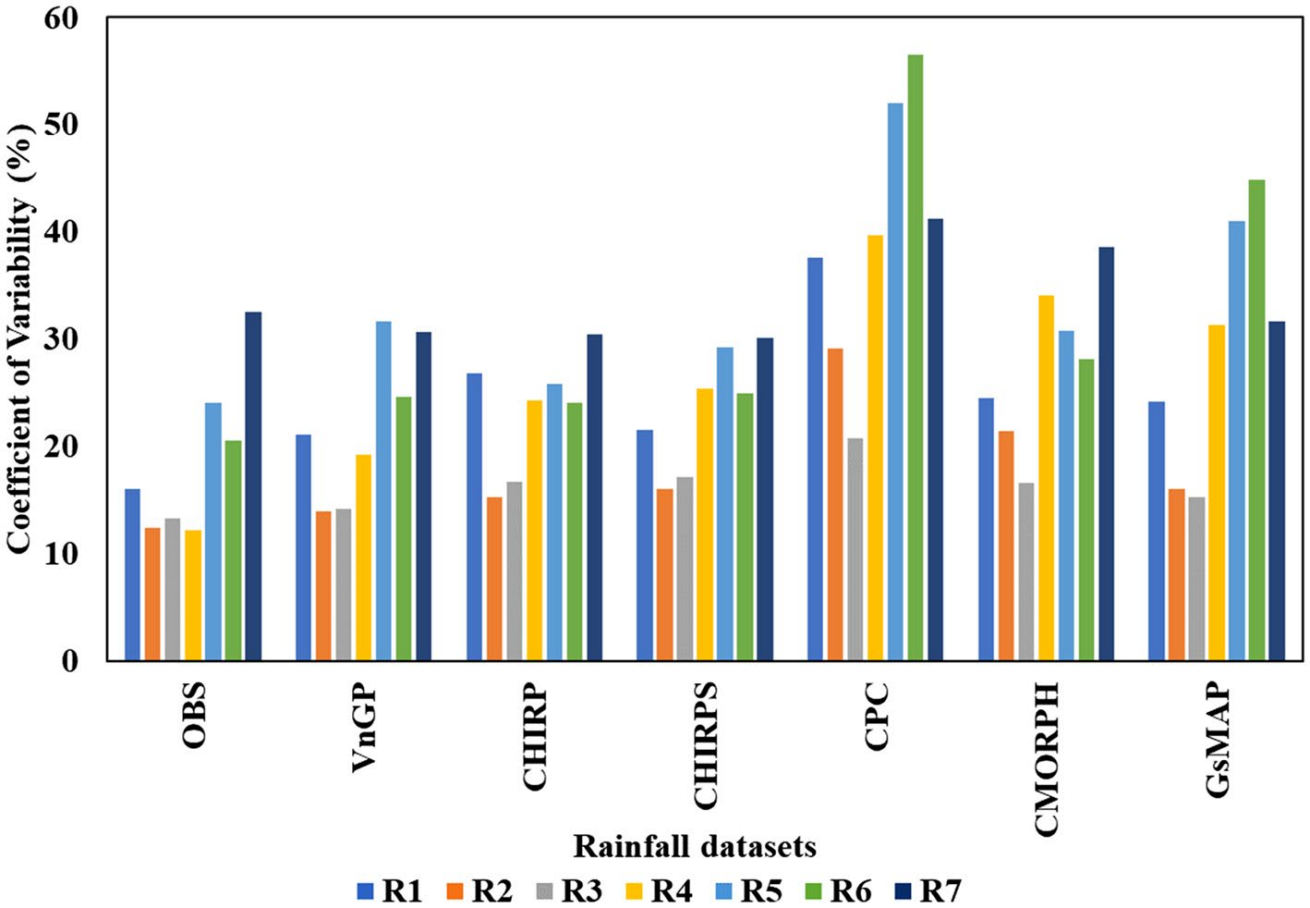
Coefficient of Variation (CV %) in maize observed (GSO maize yield data) and simulated yields with different rainfall datasets.

**Figure 7:**
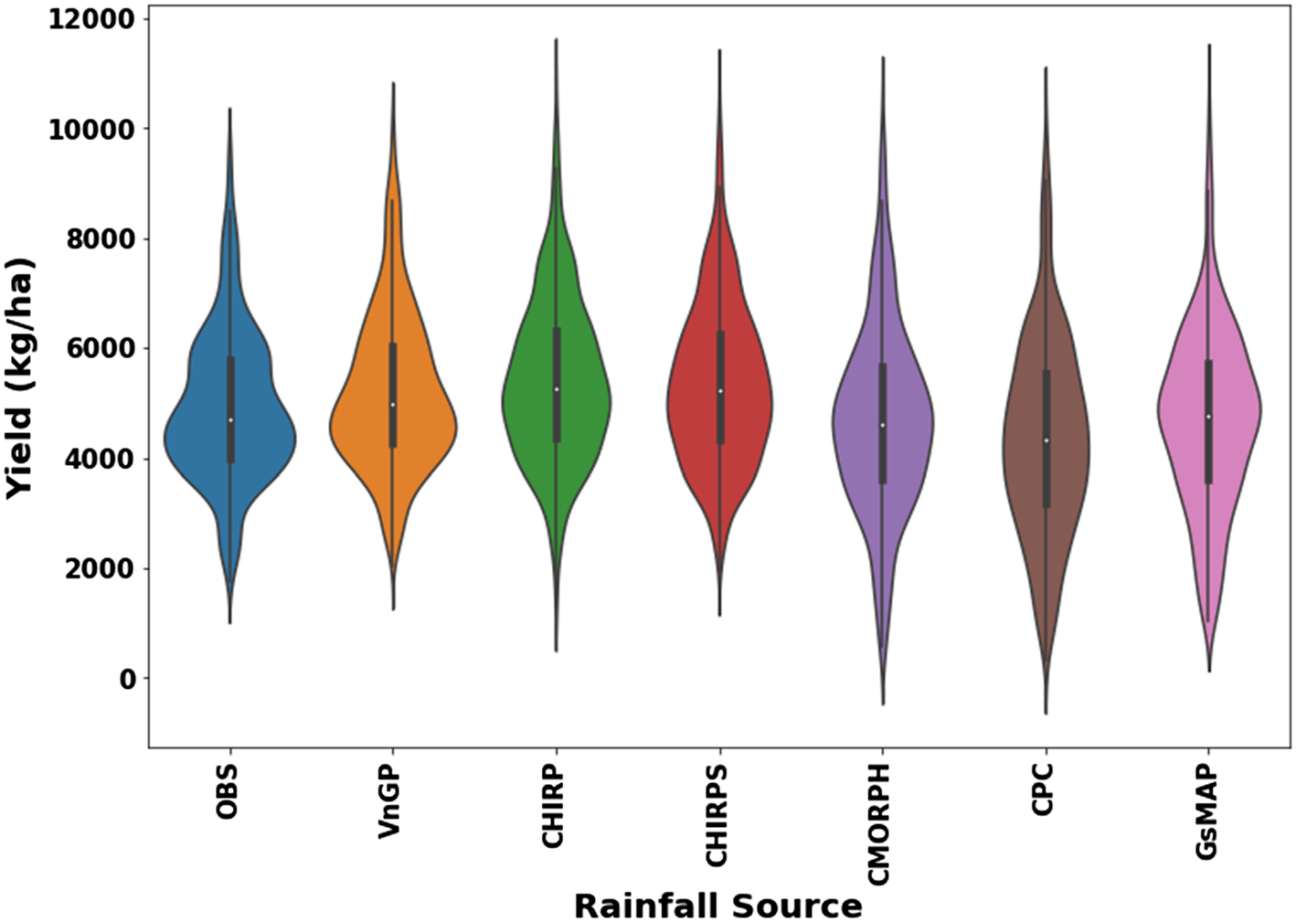
Productivity of simulated maize yields with different rainfall datasets over Vietnam.

The effect of different rainfall datasets on CERES-Maize simulations are displayed in Fig. 5. CERES-Maize simulations during the period 2001–2010 were compared with observations of all seasons and sites covered in the study. The predictive capacity of the crop simulation model at the regional level in Vietnam against GSO maize yield data exhibit a good agreement for observed rainfall (VnGP) followed by CHIRPS, CHIRP, CMORPH, GsMAP, and CPC. However, the mean yield is systematically overestimated across all rainfall datasets which is a common shortfall in many crop modeling studies. The inter-annual variability of simulated yields can be more important than mean yields. Therefore, besides average grain yields over the simulation period, standard deviation and coefficient of variation (CV%) are computed to understand the year-to-year variability. Overall, the VnGP simulated maize yields provided similar results and showed higher yield variability compared with the observed yields. Both CHIRP and CHIRPS illustrated similar trends in CV as VnGP simulated maize yields. CMORPH, CPC, and GsMAP exhibited a higher CV, maize yields simulated using CPC rainfall displayed the highest CV across all the rainfall regions (Fig. 6). The metrics (RMSE, RRMSE, MAE, RMAE, correlation coefficient (r), and Willmott D index) are summarized in Table 3, which quantifies that maize yields modeled with VnGP rainfall exhibited lowers RMSE (536 kg/ha; averaged across 7 rainfall regions) value across seven rainfall zones with the highest RMSE of 704 kg/ha at R5 and lowest RMSE of 384 kg/ha at the R3 region of Vietnam. Both CHIRP and CHIRPS demonstrated similar trends across Vietnam rainfall regions with different magnitude, CHIRP/S RMSE across maize growing regions of Vietnam is 737 and 714 kg/ha with a highest RMSE of 966 and 964 kg/ha at R7 and the lowest RMSE of 493 (CHIRP) and 449 (CHIRPS) at R1 and R2 respectively. CMORPH exhibited an average of 878 kg/ha RMSE for the seven rainfall regions with the least RMSE was observed at R2 (531 kg/ha) and underestimated maize yields at R5 and R7 where the estimated RMSE values are more than 1000 kg/ha. CPC rainfall is underestimated across the region and as a result, the maize yields simulated were underestimated, the overall RMSE value for the maize growing region is 950 kg/ha, with the highest RMSE of 1247 kg/ha at R5 and the lowest RMSE of 579 kg/ha at R3. GsMAP maize yields evaluation indicates that overall, the RMSE value for the maize growing regions is 816 kg/ha with the highest RMSE value of 1361 kg/ha observed at R4 and the lowest RMSE value of 484 kg/ha is at R2. There was a good agreement between observed yields and VnGP maize yields as the model predicted the grain yields of maize adequately with NRMSE of 11% and a d-value of 0.90 indicating that model was able to simulate maize grain yield across the study region. Both CHIRP/S rainfall simulated grain yields are in good agreement with observed yields except at R5 with NRMSE of 29% with a corresponding d-value of 0.7. Grain yields simulated using CMORPH rainfall exhibited a fair relationship with observed yields, the average NRMSE values for the seven rainfall regions is 18%, at R4 and R5 zones the NRMSE values are 21 and 26% indicating poor estimation of maize grain yields. Simulated maize yields with CPC rainfall also fall under the fair agreement category as the overall, NRMSE values for the maize growing region is 20% with a poor agreement at R4, R5, R6, and R7. The performance of different rainfall datasets in simulating maize yields across Vietnam illustrated that VnGP and CHIRP/S tend to overestimate median values and upper adjacent values. While CMORPH, CPC, and GsMAP underestimated median yield values with higher upper adjacent values as well as lower adjacent values as presented in Fig. 7.

**Table 3:** Descriptive statistics showing the performance of CERES-Maize for in reproducing historical maize yields driven with different rainfall datasets.

Rainfall source	Rainfall zone	RMSE	RRMSE	MAE	RMAE	Correlation coefficient	Willmott d index
VnGP	R1	546.7	0.148	407.6	0.112	0.82	0.84
	R2	442.0	0.099	338.1	0.078	0.80	0.83
	R3	384.1	0.072	280.2	0.055	0.91	0.95
	R4	439.6	0.102	335.8	0.083	0.85	0.88
	R5	704.0	0.137	469.5	0.099	0.88	0.93
	R6	647.9	0.123	511.6	0.105	0.91	0.93
	R7	587.7	0.113	458.1	0.106	0.97	0.98
CHIRP	R1	493.0	0.133	404.5	0.113	0.81	0.85
	R2	499.8	0.112	395.3	0.091	0.79	0.77
	R3	705.3	0.131	516.0	0.107	0.73	0.80
	R4	826.3	0.191	609.4	0.144	0.68	0.69
	R5	864.7	0.305	1253.4	0.285	0.67	0.66
	R6	810.1	0.154	639.0	0.126	0.88	0.90
	R7	966.2	0.185	739.7	0.168	0.90	0.93
CHIRPS	R1	455.7	0.123	347.9	0.096	0.80	0.88
	R2	449.0	0.101	346.9	0.080	0.79	0.82
	R3	641.2	0.119	505.1	0.103	0.80	0.83
	R4	826.3	0.191	609.4	0.144	0.68	0.69
	R5	817.8	0.296	1248.9	0.277	0.67	0.70
	R6	848.6	0.180	725.3	0.145	0.85	0.86
	R7	964.2	0.185	742.3	0.170	0.89	0.93
CMORPH	R1	623.9	0.168	469.7	0.132	0.71	0.78
	R2	531.2	0.119	402.8	0.092	0.72	0.81
	R3	749.7	0.138	630.3	0.122	0.69	0.66
	R4	904.9	0.208	799.1	0.183	0.71	0.58
	R5	1320.7	0.256	1179.2	0.248	0.67	0.57
	R6	935.2	0.176	688.4	0.134	0.71	0.68
	R7	1081.5	0.205	810.2	0.164	0.76	0.72
CPC	R1	605.0	0.163	424.3	0.121	0.65	0.79
	R2	623.6	0.140	491.7	0.111	0.59	0.73
	R3	579.1	0.108	459.2	0.095	0.79	0.65
	R4	1161.3	0.269	931.9	0.231	0.67	0.65
	R5	1246.6	0.243	926.8	0.192	0.59	0.52
	R6	1223.0	0.232	857.1	0.163	0.56	0.78
	R7	1208.0	0.232	895.9	0.187	0.85	0.89
GsMAP	R1	506.4	0.137	418.0	0.115	0.81	0.86
	R2	483.5	0.109	393.5	0.089	0.68	0.79
	R3	579.1	0.108	459.2	0.095	0.69	0.65
	R4	1361.3	0.269	931.9	0.231	0.67	0.65
	R5	949.6	0.185	730.2	0.150	0.59	0.68
	R6	677.2	0.129	445.3	0.091	0.78	0.73
	R7	1158.2	0.184	760.3	0.170	0.87	0.73

## Discussion

In this article, we have presented the results focusing on the assessment of the performance of five satellite rainfall estimates over Vietnam. The dataset presents a comparison of rainfall over Vietnam at national, rainfall regions, and pixel-level concerning magnitude, agreement, occurrence consistency, and spatial distribution. The comparison of these different products thus offers a better understanding of their weakness and robustness. Most of the products displayed higher bias in the terrain of higher elevation and coastal regions. On a national scale CHIRP/S was overestimated during the study period by 80 to 150 mm/year, however, CHIRP/S able to capture the inter-annual variability. CHIRP/S overestimates at R3 (241 mm/ year), R4 (430 mm/year), and R6 (558 mm/year), these differences are due to complex orographic rainfall produced by great elevation differences^45,46^. The interaction of the equatorward cold airflow of these continental tropical easterlies with land orography in Southeast Asia produces a large amount of rainfall in the windward areas of central Vietnam (R6). Comparison of validation statics (RMSE, MAE, and Pearson correlation coefficient) over Vietnam at national and rainfall regions showed that the performance of the satellite products varies considerably from one rainfall region to the other. This could be due to the complex climate of the region described in section 2.0. In many cases, there are significant differences observed across rainfall regions. For instance, CPC and GsMAP have stark differences across the rainfall regions region (Fig. 2) during both annual and JJA seasons. Both the satellite products failed to capture rainfall amounts over mountainous and coastal regions (R5) where most of the rainfall comes from the warm clouds. Previous studies have stated that the performance of satellite rainfall products was quite poor in high-altitude regions because of its ability to detect warm orographic rainfall^41,42,47^. The performance of satellite products is consistent with observed rainfall when aggregated at national and rainfall region levels with minor differences. The performance of these products increased from daily to monthly, seasonal and annual time scales. A poor performance in the mountainous area is notable for satellite estimates. These results are in agreement with other studies^47–49^, which indicates that satellite rainfall products have challenges in estimating orographic rainfall in complex topography.

All products presented good performance for occurrence consistency, with a mean POD rate are higher than 60%, except for GsMAP and CMORPH. False rates and misses are relatively uniform across the five products. CPC, CMORPH, and GsMAP displayed high false events, which may be due to their low spatial resolution or lack of gauge correction. The capacity of satellite rainfall products to capture precipitation occurrence events may also be impacted by the revisit cycle of the sensors. High elevation dependency is noticed for CPC, CMORPH, and GsMAP according to Fig. 2. While CHIRP and CHIRPS products well captured the elevation-affected precipitation in the north (R1) and central highlands (R6). The low spatial resolution, free-evaluation correction, and free-gauge can be attributed to having a low correlation with the topography for the three products (CPC, CMORPH, and GsMAP).

Based on the above discussion, we can see that two of the five satellite-based products, CHIRP, and CHIRPs, performed the most consistently for the observed data across different rainfall regions of Vietnam. CHIRP estimates precipitation based on infrared Cold Cloud Duration observations while, CHIRPS estimates precipitation also merges multiple data, such as tropical rainfall measuring mission multi-satellite precipitation analysis (TMPA), CMORPH, and global geosynchronous TIR archives, etc^22^. CHIRP/S has shown a good correlation with observations in East Africa^50^, and South Asia^51^. CHIRPS were strongly recommended for agricultural and hydrological research because of their excellent performance. Relatively weak spatial distribution of CMORPH rainfall can be attributed for, primarily a microwave-based product and it estimates precipitation largely based on scattering by ice aloft^52^, which might lead to underestimation of rain. A weak correlation is also reported by^53^.

Accurate and high-resolution rainfall information plays a vital role in the impact assessment of agricultural systems using biophysical processes-based crop simulation models. In this study maize yields simulated with different rainfall, sources are typically used to evaluate the performance of estimated rainfall products across a wide range of environments in Vietnam. CERES-maize yields simulated across major maize growing environments using observed rainfall and five satellite rainfall products showed that the modeled maize yields are systematically higher than reported maize yields across Vietnam. Crop simulation models simulate attainable crop yields based on the local environmental conditions ignoring biotic stress and other on-farm practices accurately^54–56^. Different layers of errors in the simulated crop yields are from inaccurate representation of soil information, climate, on-farm crop management practices. In Vietnam, the CERES-maize model simulated with observed climate conditions represents quite well the crop yields year-to-year variability from 2001 to 2010. Satellite estimated rainfall datasets used for crop simulation models, we found large biases might be introduced in crop simulations either through water stress or through nitrogen availability. Maize yield modeled with estimated rainfall datasets illustrated higher inter-annual variability particularly, for CPC and GsMAP, which tend to underestimate rainfall volumes across Vietnam. These biases depend on satellite estimated errors in cumulative rainfall totals during crop-growing periods on the temporal distribution. This spatial interpolation of daily rainfall is likely to introduce biases in rainfall frequency (overestimated) and intensity (underestimation of high rainfall) as shown by^57^. CHIRP/S outperformed in reproducing maize yields during the study period, while CMORPH, CPC, and GsMAP displayed the highest biases in simulated maize yields. Satellite products with positive biases resulted in over-estimated maize yields and negative biases underestimated maize yields in the study region.

An important limitation of this study is we are comparing simulated crop yields driven by estimated satellite rainfall data at different spatial resolutions. Interpolated satellite rainfall products contain errors due to the interpolation procedure. Another limitation of this study is the sowing window which is triggered when given criteria are met such as, 25 mm received in 10 consecutive days. The capacity of the satellite products in estimating accurate rainfall events that prompted sowing may be crucial for studies that used estimated gridded precipitation datasets for risk characterization, developing suitable adaptation packages for climate variability.

## Summary and conclusions

The dekadal and monthly rainfall estimates from the five satellite-based products better correlate with the observed data with significant correlation coefficient than the daily data across rainfall zones. The national level dekadal rainfall data also revealed highly significant correlation with the observed data in the order of CMORPH, CHIRP, CHIRPS, CPC and GsMAP. Also, daily rainfall data at national level has significant correlation coefficient between satellite-based rainfall and observed rainfall hierarchically in the order CPC, CMORPH, CHIRP, CHIRPS and GsMAP. The validation exercise of satellite rainfall revealed interesting spatial patterns in the performance of the different satellite rainfall products. The evaluation results indicate that the satellite rainfall products have challenges in estimating rainfall totals in mountainous and coastal regions. However, this does not specify that all mountainous regions have the same behaviour. Rainfall variability in specific terrain conditions need to be studied using lapse rate in rough terrains and atmospheric pressure differences causing cyclonic disturbances. The performance of satellite rainfall products over the Northern parts of the Vietnam (R1) region failed to capture the spatial variability, while in the central highlands, CHIRPS and CMPORPH captured spatial variability. Both CHIRP and CHIRPS performed better than CPC, CMORPH, and GsMAP. CPC and GsMAP rainfall products underestimated rainfall totals over Vietnam, CMORPH performance is better in the central and southern region of Vietnam and overestimated rainfall totals in the northern region. As expected, CHIRPS performance is much better than CHIRP due to bias adjustment using station data, this demonstrated that the performance of satellite rainfall products could be significantly improved with a mean bias correction using station data. The bias adjustment using station data could also help to improve estimated rainfall products to make them temporally more homogenous as these inputs may not change over time unless there is a significant reduction or increasing trend in rainfall totals for the given region.

Despite some crudity in satellite estimated rainfall used in the study, there appears a potential opportunity in coupling crop simulation models with satellite rainfall estimates of near real-time for crop production estimates. This approach of obtaining crop production estimates in data-limited environments facilitates such yield projections that are required for planning and decision making. Production/yield estimates using satellite rainfall data require several areas where further improvements are needed. The soil database needs further refinement to accurately represent soil properties spatially in the cropping areas particularly, with regard to soil initial conditions. The quality of input data required by crop simulation models can be improved such as accurately representing field conditions and crop management practices employed. The daily weather data required for crop simulation models to simulated crop yields are perhaps most critical. The scarcity of operational rain gauge networks and difficulty to access data in near real-time poses significant challenges for operational yields forecasting, crop risk characterization, development of location-specific crop suitable adaptation packages, etc. Rainfall monitoring using satellites provided a good opportunity for developing a yield forecasting mechanism using dynamical crop simulation models. A wide range of satellite rainfall products is now easily available in near real-time. However, these operational rainfall products exhibit uncertainty in estimating rainfall totals and rainy days, and the propagation of such uncertainty through crop simulation models needs to be addressed before using estimated rainfall for yield prediction. In this study, we showed that the use of raw satellite rainfall estimates can introduce large uncertainties in crop modeling experiments. The misrepresentation of seasonal rainfall amounts, distribution may significantly affect the availability of nitrogen, water-stress and lead to poor crop yield estimates. Depending on the local conditions an overestimate or underestimate of rainfall totals during crop growing periods can lead to either overestimated or underestimate crop productivity. The tested five satellite rainfall products displayed large uncertainties in yield prediction, high-resolution bias-adjusted products such as CHIRPS displayed good skill in reproducing historical yields, this product appears to be most suitable for crop yield estimation studies in Vietnam.

## Study region and data

**Figure 8:**
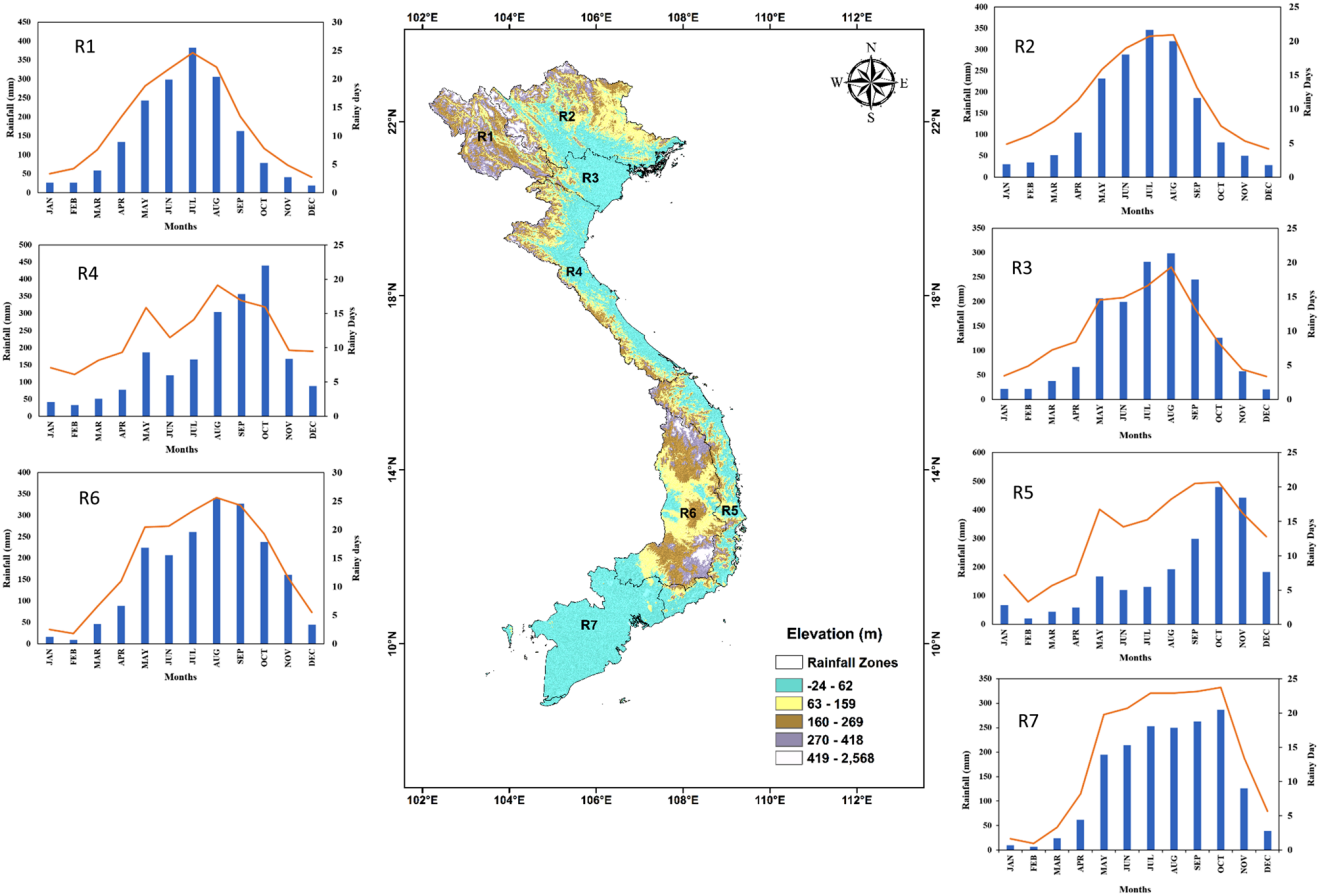
Map displaying seven rainfall regions in Vietnam and its complex topography (elevation). Rainfall regions: **R1** (North West), **R2** (North East), **R3** (Red River Delta (RRD), **R4** (Central Region of Northern Mountains), **R5** (South Central Coast), **R6**: (Central Highlands) and **R7** (Mekong River Delta). These maps were generated using ArcGIS 10.8.1 (https:// www. arcgis. com/ index. html) and observed gridded rainfall data set (http://danida.vnu.edu.vn/cpis/en/content/gridded-precipitation-data-of-vietnam.html).

Vietnam is situated in the tropical monsoon zone which is close to the typhoon center of the western pacific and falls under the most disaster-prone countries. The region has the most complex topography, elevation varies from sea level to Fansipan mountain in Lao Cai Province in Vietnam at 3144 m. The monsoon system and tropical disturbances in the Intertropical Convergence Zone (ITCZ) influences rainfall regimes in Vietnam. Monsoon over Vietnam is regulated by the southwest summer and the northeast winter monsoons. Northern and southern Vietnam receives maximum rainfall during the summer (June-July–August, JJA), September–October-November (SON) season contributes maximum rainfall over Central Vietnam. In some parts of Central Vietnam, the annual rainfall can reach between 3600 and 4000 mm^58^. The climate of the region is strongly influenced by El Niño Southern Oscillation (ENSO) cycles^44,59^. The interactions between the regional climate and global atmospheric phenomenon are complex, for instance, different phases of ENSO have different impacts during a different season and over different regions of Vietnam. Several studies have been conducted on the interannual variability of rainfall over Vietnam and its association with ENSO events^60–63^. The complex topographic and climate of Vietnam offer both opportunities as well as challenges to validate satellite rainfall products. The diverse conditions over Vietnam provide an opportunity to test the performance of satellite products over different rainfall regimes. Generally, TIR-based rainfall products have poor estimations in orographically induced rainfall^47^ due to rain received from clouds of higher temperatures than the threshold used by satellite algorithms. In this study, we applied the same climate sub-regions as^58^, displayed in Fig. 8 with marked subregions (R1 to R7). The topographical features over Vietnam, taken from the SRTM (Shuttle Radar Topography Mission) dataset, are displayed in Fig. 8. The sub-regions R1, R2, and R6 have high topography while R3 and R7 are the delta areas with very low elevation. R1 (Northwest), R2 (Northeast), and R3 (Red River Delta) displayed similar rainfall distribution, the wet season in the three sub-regions is between May and October. Rainfall regions R4 (North Central) and R5 (South Central Coast) wet season starts from May and extends to November/December with peak rainfall received during Sept/Oct/Nov while in R6 (Central Highlands) and R7 (Mekong River Delta) receives rainfall during May to October.

## Reference data

The VnGP gridded dataset^64^ is used to evaluate the satellite rainfall products. This daily gridded rainfall data is available at two different spatial resolutions (10 × 10 km and 25 × 25 km) from 481 gauges' rain-gauge data from the period 1980–2010. The study by^64^ has compared VnGP with APHRODITE (Asian Precipitation – Highly-Resolved Observational Data Integration Towards Evaluation) and station data. Considering the number of rain gauges used in developing the gridded precipitation data, the VnGP is the best-gridded data over Vietnam. One of the limitations of the VnGP dataset is that it is not updated regularly and data after 2010 is still under development as per the communication with Phan Van Tan. Therefore, the VnGP dataset is utilized in this study as the reference gridded observation dataset.

### Satellite data

#### CHIRP/S data

CHIRPS is a high-resolution quasi global gridded rainfall dataset starting from 1981 to the current period and is developed to deliver a reliable and complete dataset that can be used to understand trends in rainfall variations and drought monitoring over time. CHIRPS is developed at 0.05° and 0.25° resolution by combining satellite estimates with in-situ station data^22^. The CHIRP/CHIRPS product used in this study were downloaded from ftp://chg-ftpout.geog.ucsb.edu/pub/org/chg/products/CHIRPS-2.0/global_daily/.

#### CMORPH data

CMORPH^65^ provides global precipitation data at 30-min temporal resolution and 0.073 degrees (∼8 km) spatial resolution. In this technique, two images of infra-red (IR) at 30-min time intervals are used to compute the moving trajectory of the cloud in time and half-hourly precipitation rate is estimated from Passive Microwave (PMW) propagated along the moving vector. Thus, allows a decrease in sampling error by incorporating time-weighted linear interpolation. The Precipitation rate during the gap between IR and PMW is morphed to estimate the shape and intensity of the precipitation^65^. This allows combining the superior retrieval accuracy of passive mode estimates and the higher temporal and spatial resolution of IR data^47^. These data are downloadable from: ftp://ftp.cpc.ncep.noaa.gov/precip/global_CMORPH/.

#### CPC

The National Oceanic and Atmospheric Administration’s (NOAA) Climate Prediction Center (CPC) Unified Precipitation Project produced high-quality precipitation products over land globally^66^. The CPC rainfall data used in this study covers the global land on a coarser resolution of 0.5° × 0.5°. The goal of the CPC unified project is to develop improved quality by combining all information sources available at CPC and by taking advantage of the optimal interpolation (OI) objective analysis technique. The daily precipitation has been constructed over the global land areas using gauge data reports from 30,000 stations collected from multiple sources such as Global Telecommunication System (GTS), Cooperative Observer Program (COOP), and other national and international agencies. In this study, the gridded daily CPC rainfall estimates performance is evaluated.

#### GSMaP

Japan Science and Technology Agency (JST) and Japan Aerospace Exploration Agency (JAXA) support the GSMaP project^67–69^. GSMaP integrates both PMW and IR sensors to provide global precipitation estimates at high temporal (hourly) and spatial resolution (0.1°) that covers quasi global (60°N -60°S). In this study, we used the standard product version GSMaP_MVK, which is produced based on the Kalman filter model that improved precipitation rate based on the atmospheric vector derivative of two successive infrared images^70^.

### Evaluation of Satellite estimated rainfall (SER)

The foremost focus of this validation work is to assess the performance of the different satellite rainfall estimates over Vietnam and its applicability for impact assessment studies. The performance of these products is evaluated using the VnGP gridded rain gauge dataset. This section describes the details of the methodology used to evaluate the satellite rainfall products at different spatial and temporal scales.

#### Approach

Validation was done both at regional and national levels. Satellite rainfall estimates at each grid point were compared to the corresponding VnGP (0.1°) grid for annual and seasonal totals and rainy days. An overview of the reference (VnGP) and satellite gridded datasets are provided in Table 4. Because of inconsistency in data length and spatial resolution, the analysis is performed for the period 1981–2010. Evaluation of satellite rainfall estimates for crop modeling is performed for the period 2001–2010. The spatial patterns of five satellite products were also compared with VnGP data at daily, dekadal and seasonal time scales. Spatial variability of the rainfall products is calculated for each grid point and aggregated to climate sub-regions in Vietnam.

**Table 4:** Summary of satellite rainfall products used in the study.

Data source	Spatial resolution	Temporal Resolution	Period	References
VnGP	0.10	daily	1981–2010	Nguyen et al.^64^
CHIRP	0.05	daily, pentadal, monthly	1981-Present	Funk et al.^22^
CHIRPS	0.05	daily, pentadal, monthly	1981-Present	Funk et al.^22^
CPC	0.05	3 hourly, daily	1979-Present	Xie et al.^71^
CMORPH	0.25	daily	2002-Present	Joyce et al.^65^
GsMAP	0.10	1 hourly,3 hourly, daily	2000-Present	Okamoto et al.^72^

#### Evaluation method

Different metrics are used for the validation and verification of satellite rainfall products. Evaluating of the performance of satellite rainfall products at a daily time scale is focused on whether the products can detect the occurrence of rainfall. The different categorical statistics are used in this study are the Probability of Detection (POD), False Alarm Ratio (FAR), Probability of False Detection (POFD), and Heidke Skill Score (HSS). The statistical skill scores are based on a likelihood table (Table 5), where A, B, C, and D represent hits, false alarms, misses, and correct negatives, respectively, and are given as:

**Table 5:** Contingency table for comparing rain gauge measurements and satellite precipitation estimates. The rainfall threshold used is 1.0 mm.

	Gauge ≥ threshold	Gauge < threshold
Satellite ≥ threshold	A (hits)	B (false alarm)
Satellite < threshold	C (miss)	D (correct rejection)

The selected satellite rainfall products considered in this study are at different spatial scales, hence the comparison is performed using station data, the point-pixel evaluation is performed, meaning each rain gauge station data is compared to the nearest satellite pixel. The nearest rainfall pixel for each satellite rainfall product to the station is identified using nearest neighbor algorithm in ArcGIS 10x.

The skill of the satellite products in detecting the occurrence of rainfall is assessed with POD, which represents the ratio of a correct number of rainfall occurrences to the total number of reference rainfall events. A high POD indicated that the estimated rainfall product exhibits a good agreement to correctly perceive rain events.

1 $$POD = \frac{A}{A + C}$$

POFD identifies a fraction of non-rainy-day events that are identified as rainy-day events by satellite estimated rainfall products. The POFD displays how many non-rain events missed predictions.

2 $$POFD = \frac{B}{B + D}$$

While, the false detections are assessed with FAR, which represents the ratio of the number of false occurrences of precipitation to the total number of satellite precipitation occurrences.

3 $$FAR = \frac{B}{A + B},{\text{ and}}$$

The HSS statistic measures the skill of satellite estimated rainfall that compares the proportion of correct estimates while accounting for matches due to random chances. An HSS of 1 represents a perfect estimation, and an HSS of 0 denotes poor estimation.

4 $$HSS = \frac{{2\left( {AD - BC} \right)}}{{\left( {A + C} \right)\left( {C + D} \right) + \left( {A + B} \right) \left( {B + D} \right)}}$$

The rainfall threshold used in this study for identifying rainy days is 1 mm/day. The rainfall threshold may increase the frequency of rainfall occurrence by satellite data relative to observed rainy-day frequency (VnGP). The satellite rainfall products were also evaluated using continuous statistics to measure the accuracy of variables such as rainfall amount and intensity against VnGP using Correlation Coefficient estimates, bias, Mean Absolute Error (MAE), Root Mean Square Error (RMSE), and Nash–Sutcliffe Coefficient of Efficiency (NSCE) which offer an insight into the skill of the products in estimating rainfall amounts (Table 6). Pearson Correlation Coefficient estimates the linear relationship between the observed and estimated values where r =  + 1 is an indication of a perfect linear relationship between observed and estimated rainfall. The correlation coefficient estimates are computed between station gauge data and the nearest satellite rainfall pixel at daily and dekadal (10-day) scale. The RE is the ratio of obsolete error to observed measurements and is used as a measurement of precision. NSCE is used to assess the performance of the satellite in predicting observed precipitation with values ranging from − ∞ to 1, where NSE = 1 indicates a perfect match between estimated and observed rainfall. The equations to calculate the above-mentioned indices are listed below. In addition to these commonly used comparison statistical metrics, the cumulative distribution function (CDF) for the number of rainfall events at the daily time scale was used to evaluate rainfall totals.

**Table 6:** The list of statistical evaluation indices to evaluate quantitative rainfall estimates.

Evaluation indexes	Formulas	Description	Perfect value
Correlation coefficient (CC)	$CC = \frac{{\left( {O_{i} - \overline{O}} \right)\left( {S_{i} - \overline{S}} \right)}}{{ \sqrt {\left( {O_{i} - \overline{O}} \right)^{2} \left( {S_{i} - \overline{S}} \right)^{2} } }}$	S_i_ and O_i_ are the satellite and observed values; $\overline{S}$ and $\overline{O}$ are the mean values of S_i_ and O_i_ n is the number of samples	1
Mean error (RE)	$ME = \frac{1}{N}\sum \left( {S_{i} - O_{i} } \right)$		0
Mean absolute error (MAE)	$MAE = \frac{1}{N}\sum \left| {\left( {S_{i} - O_{i} } \right)} \right|$		0
Root mean square error (RMSE)	$RMSE = \sqrt {\mathop \sum \limits_{i = 1}^{n} \frac{{\left( {S_{i} - \overline{S}} \right)^{2} }}{n}}$		0
Nash–Sutcliffe coefficient of efficiency (NSCE)	$$NSCE = 1 - \mathop \sum \limits_{i = 1}^{n} {\raise0.7ex\hbox{${(S_{i} - O_{i} )^{2} }$} \!\mathord{\left/ {\vphantom {{(S_{i} - O_{i} )^{2} } {\mathop \sum \nolimits_{i = 1}^{n} \left( {O_{i} - \overline{O}} \right)^{2} }}}\right.\kern-\nulldelimiterspace} \!\lower0.7ex\hbox{${\mathop \sum \nolimits_{i = 1}^{n} \left( {O_{i} - \overline{O}} \right)^{2} }$}}$$		1

### Biophysical model: CERES-Maize model

The DSSAT Cropping System Model (CSM) is a process-oriented model which is capable of modeling long-term crop simulations under different environmental conditions^73,74^. The CERES-Maize model is part of a suite of crop models available in DSSAT v4.7, is used to simulate the daily maize growth until physiological maturity and harvest stage. CERES-Maize model required inputs are daily weather data (maximum and minimum temperature, solar radiation, and precipitation), soil (profile wise soil physical and chemical properties), crop management data includes amount and method of residues application, planting dates, irrigation, fertilization, etc., and the cultivars genetic coefficients. Profile-wise soil data used in this study is SoilGrids1km developed by ISRIC, in collaboration with several international agencies^75^. SoilGrids1km offers properties of soil profiles at six depth intervals 0–5, 5–15, 15–30, 30–60, 60–100, and 100–200 cm. The soil properties include sand, silt, and clay fractions (%), bulk density (kg m − 3), pH, soil organic carbon (g kg − 1), cation exchange capacity (cmol kg − 1), coarse fragments (%). To simulate the growth, development, and yield of major maize growing environments of Vietnam, we used International Food Policy Research Institute (IFPRI), Spatial Production Allocation Model (SPAM) to identify plausible maize crop distribution (IFPRI, 2019). In the present study, the physical areas of irrigated (SPAMir-mz) and rain-fed maize (SPAMrf-mz), were extracted spatially. For the yield simulations, we selected three hybrid maize cultivars (SX2010, SX5012, and LVN47) widely cultivated across Vietnam under different environmental conditions. The three calibrated and validated maize cultivars^76^ were used to simulate maize yields across Vietnam driven by different satellite estimated rainfall products. Planting window, cultivar, amount of fertilizer, residual application amounts for each province are collected from published and unpublished records of the Vietnam Academy of Agricultural Sciences (VAAS). Further details can be accessed from http://ngo.gap-vietnam.com/sanxuatngotrenthegioivavietnam.php. For model validation, historical maize yields reported by the General Statistics Office (GSO) of Vietnam at the province level for the period 2001–2010 were used^29^. A simple linear regression analysis was performed on historical maize yields reported at the province level to identify technology trend, this method is widely to detrend crop yields in the past studies^77,78^ used to detrended reported maize yields for each province and then compared with CERES-maize simulated yields. The CERES-Maize simulated yields were evaluated using Correlation Coefficient (CC), Mean Absolute Error (MAE), normalized MAE, Root Mean Square Error (RMSE), normalized RMSE, and d-index which offer an insight into the skill of the products in simulating maize yields spatially (Table 7).

**Table 7:** The list of statistical evaluation indices to evaluate modeled maize yields.

Evaluation indexes	Formulas
Normalized RMSE (%)	$= \left( {\frac{{absolute{ }RMSE}}{{\overline{O}}}} \right) \times 100$
d-index)	$1 - \left[ {\frac{{\mathop \sum \nolimits_{i = 1}^{n} \left( {P_{i} - O_{i} } \right)^{2} }}{{\mathop \sum \nolimits_{i = 1}^{n} \left[ {P_{i}^{^{\prime}} + O_{i}^{^{\prime}} } \right]^{2} }}} \right]$
